# Supplementary material for: Formulation and Evaluation of Chitosan/NaCl/Maltodextrin Microparticles as a Saltiness Enhancer: Study on the Optimization of Excipients for the Spray-Drying Process
Source: Polymers (Basel). 2021 Dec 9;13(24):4302. doi: 10.3390/polym13244302 (PMC8706731; doi:10.3390/polym13244302)
Supplement: Supplementary file 1 [file polymers-13-04302-s001.zip › polymers-1463227-supplementary.pdf]

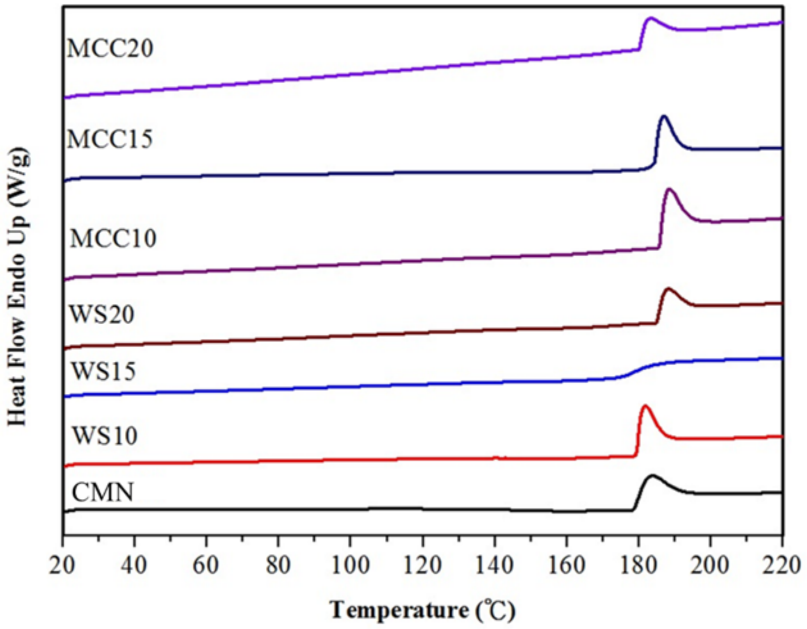

Figure S1. The DSC curves of chitosan/NaCl/maltodextrin microparticles. CNM indicates the formulation with only maltodextrin as the excipient. MCC indicates the for-mulations with maltodextrin and micro-crystalline cellulose as the excipients. WS indicates the formulations with maltodextrin and waxy maize starch as the excipients. The compositions of each formulation are the same as in Table 1.
